# A Multi-Stage Progressive Network with Feature Transmission and Fusion for Marine Snow Removal

**DOI:** 10.3390/s24020356

**Published:** 2024-01-07

**Authors:** Lixin Liu, Yuyang Liao, Bo He

**Affiliations:** 1 Institute of Deep-sea Science and Engineering, Chinese Academy of Sciences, Sanya 572000, China; liaoyy@idsse.ac.cn (Y.L.); hb2281230012@gmail.com (B.H.); 2 University of Chinese Academy of Sciences, Beijing 100049, China

**Keywords:** marine snow, underwater image processing, multi-stage, deep learning

## Abstract

Improving underwater image quality is crucial for marine detection applications. However, in the marine environment, captured images are often affected by various degradation factors due to the complexity of underwater conditions. In addition to common color distortions, marine snow noise in underwater images is also a significant issue. The backscatter of artificial light on marine snow generates specks in images, thereby affecting image quality, scene perception, and subsequently impacting downstream tasks such as target detection and segmentation. Addressing the issues caused by marine snow noise, we have designed a new network structure. In this work, a novel skip-connection structure called a dual channel multi-scale feature transmitter (DCMFT) is implemented to reduce information loss during downsampling in the feature encoding and decoding section. Additionally, in the feature transfer process for each stage, iterative attentional feature fusion (iAFF) modules are inserted to fully utilize marine snow features extracted at different stages. Finally, to further optimize the network’s performance, we incorporate the multi-scale structural similarity index (MS-SSIM) into the loss function to ensure more effective convergence during training. Through experiments conducted on the Marine Snow Removal Benchmark (MSRB) dataset with an augmented sample size, our method has achieved significant results. The experimental results demonstrate that our approach excels in removing marine snow noise, with a peak signal-to-noise ratio reaching 38.9251 dB, significantly outperforming existing methods.

## 1. Introduction

Underwater imaging technology has found widespread applications as a vital component for understanding underwater marine environments [1]. However, due to the inherent adverse conditions in underwater imaging, the captured underwater images often suffer from certain degradation [2,3,4]. Apart from common issues such as color distortion, marine snow noise in underwater images is a significant concern. Marine snow refers to tiny particles that exist in the ocean and sink to the seabed. These speckles are composed of remnants of underwater organisms, floating fecal matter, suspended sediments, and other inorganic materials [5]. They exhibit various sizes, shapes, transparency and, as they settle to the seabed, resemble snowflakes, bearing similarities to atmospheric snow, hence the name marine snow.

**Figure 1:**
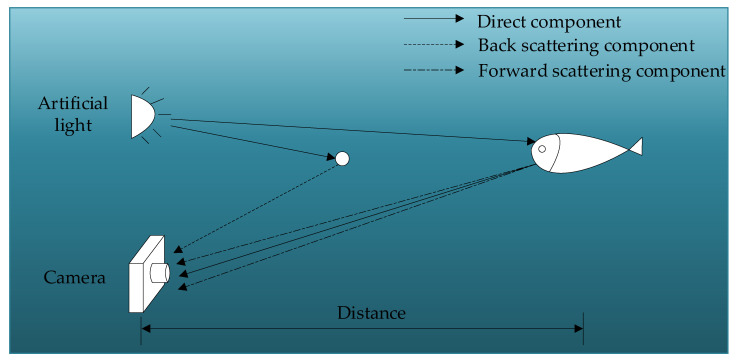
Schematic diagram of forward and back scattering. We use two different dashed lines to represent forward scattering and backward scattering. The backward scattering generated by the artificial light source on small particles can interfere with the camera’s capture of underwater images.

According to the underwater imaging model proposed by Jaffe-McGlamery [6], as illustrated in Figure 1, the artificial light is scattered by the suspended marine snow particles and enters the camera [7], which results in the appearance of bright white spots in the captured images. Due to the high brightness of these spots, they can be mistakenly recognized as real features in object detection and segmentation tasks, consequently affecting the performance of downstream tasks.

Currently, research on marine snow removal is relatively limited. Most traditional methods [8,9,10,11,12,13] treat marine snow as impulsive noise and remove it using techniques like median filtering or background modeling. However, median filtering inevitably introduces image blurring, and background modeling is restricted to fixed camera positions, with non-background parts potentially obscuring background information. In addition, some sporadic research attempts [14,15] have used deep learning methods for marine snow removal, such as processing the high-frequency components of separated marine snow using networks based on residual learning strategies or using image-to-image generation networks to eliminate marine snow. Nevertheless, the former relies on the premise of separating the high-frequency components of marine snow, and the latter has limitations in terms of pattern breakdown and challenging training.

In order to obtain higher-quality underwater images, this paper focuses on the targeted treatment of the underwater characteristics of marine snow based on the concept of multi-stage progressive restoration. We have developed the multi-stage progressive marine snow removal network (MP-MSRN) on the main structure of MPRNet [16]. The primary contributions of this study are outlined as follows:

1. We incorporate the multi-stage progressive recovery method and a feature fusion module for the marine snow removal task. This strategy not only gradually enhances the extraction of marine snow features but also ensures the full utilization of features at different stages, resulting in favorable outcomes.
2. We propose a novel skip-connection structure, named DCMFT, to ensure comprehensive feature propagation across different scales during the encoding and decoding processes, thus reducing information loss caused by downsampling.
3. We also design a new weight multi-scale adaptive loss function to accelerate the convergence speed during the network training process.

## 2. Related Work

Currently, the focus of underwater vision tasks is primarily centered around underwater image enhancement [17,18], with relatively little attention given to the removal of marine snow. Only a small portion of research is dedicated to this specific area. Based on the different methods for marine snow removal, we can roughly categorize them into two types: traditional methods and deep learning methods, which we will discuss in detail in this section.

### 2.1. Traditional Methods

Traditional methods for removing marine snow, with the median filter as a representative, treat marine snow noise as impulsive noise and use the median within the filter kernel as a replacement. Typically, traditional median filtering methods employ global processing, which may result in non-marine-snow areas’ pixels also being replaced, thereby reducing the overall image quality [19].

To overcome this issue, Fahimeh Farhadifard et al. [11] introduced the supervised median filter. They divided the filtering process into two stages: coarse filtering and fine filtering. In coarse filtering, they selected image blocks that meet certain constraints, while in fine filtering, a voting mechanism was employed to determine the removal locations, avoiding interference with object edges. Banerjee et al. [8] also proposed an adaptive median filtering method by calculating the probability of high-luminance pixels being marine snow in each image block and applying median filtering to image blocks with probabilities exceeding a threshold. However, these two methods impose strict requirements on marine snow detection, and misjudgments can lead to a decrease in image quality.

Subsequently, Fahimeh Farhadifard et al. [10], in another paper, addressed marine snow noise in continuous video frames using a background modeling approach. They selected image blocks based on previous algorithms, extracted a marine snow position mask, and used prior information about the scene to restore the scene behind the marine snow, thereby eliminating the marine snow. By contrast, Cyganek et al. [9] employed a three-dimensional median filter to address the issue of marine snow noise pixels detected in the image. The approach involves substituting the median values of adjacent layers for the identified noise pixels. Additionally, a two-dimensional frame median filter is applied to further eliminate the remaining small particles’ noise. The former is constrained by a fixed background, while the latter relies somewhat on the sparse nature of marine snow.

### 2.2. Deep Learning Methods

With the continuous development of computer vision technology, deep learning methods are increasingly being applied in the field of oceanography, including the problem of handling marine snow. Wang et al. proposed a marine snow removal network based on a residual learning strategy [20]. They used guided filters [21] to separate the white marine snow information in the high-frequency layer. Subsequently, through the processing of multiple local residual blocks, they fused it with low-frequency layer information, successfully achieving marine snow removal. However, it is worth noting that the high-frequency layer information is not entirely related to marine snow. It also contains background information, which may lead to the loss of original image information.

On the other hand, Guo et al. [14] adopted a different approach, treating marine snow removal as an image domain transformation task [22]. They used generative adversarial network (GAN) models to perform this operation, focusing on overall image transformation rather than just the marine snow’s location. In this field, CycleGAN [23] is a typical model that uses the constraint of cycle consistency to train generators between different image domains, achieving the transformation from snow-covered to snow-free conditions, as does its derivative networks [24,25,26]. However, the consistency constraint of GANs pertains to the overall image and does not focus on pixel-level details. Therefore, in image-to-image transformation, it may affect parts that are not necessary to change.

Approaching the issue from a different perspective, the elimination of marine snow falls within the realm of image restoration. Zamir et al. [16] introduced a multi-stage complementary feature processing strategy, combining an encoder–decoder and an original resolution subnetwork, achieving commendable restoration results in recovery tasks. However, the fixed nature of the multi-stage design hinders the dynamic adjustment of the network’s depth and complexity based on the degradation level. Consequently, targeted improvements addressing the micro-particle characteristics of marine snow are still necessary.

## 3. Method

**Figure 2:**
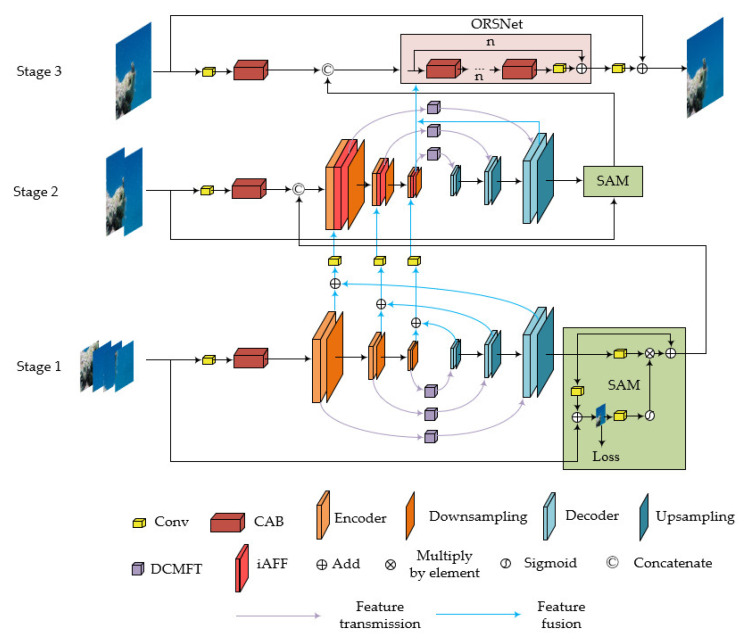
The overall framework of MP-MSRN. The entire network is divided into three stages. The network structures in the first two stages are similar, employing an encoding–decoding structure followed by the use of the SAM module for marine snow removal in this stage and passing features to the next stage. Feature fusion between the two stages is achieved using the iAFF module. In the third stage, ORSNet is introduced to preserve fine details in the output image.

In Figure 2, we present the architecture of the MP-MSRN, which comprises three main stages. In the initial stage, the network processes the input by dividing it into four sub-blocks. As the stages progress, the size of the sub-blocks gradually increases, and the number of sub-blocks decreases until the third stage, where no further sub-blocking is performed. The purpose of this design is to enable the network to learn as many details about marine snow as possible in the initial stage, while preserving the original input information for subsequent restoration operations in the final stage.

During the initial two stages, through a U-shaped encoder–decoder structure, the feature maps of marine snow are separated. Some features are fused with the encoding results of the next stage using the iAFF [27] module, while another part is used for image restoration and the enhancement of marine snow features, through a self-supervised attention module and a skip connection from the original input in this stage. Additionally, the restored image is compared to the ground truth to calculate a part of global loss, and the enhanced marine snow features are integrated with the input before the encoder–decoder in the next stage.

Finally, in the third stage, an original resolution sub-network (ORSNet) is employed to generate high-resolution features, ensuring that the final output image includes fine details. 

In the following sections, we will provide a detailed description of the composition details of each module, including the DCMFT, iAFF, supervise attention module (SAM), ORSNet, and loss functions.

### 3.1. DCMFT

**Figure 3:**
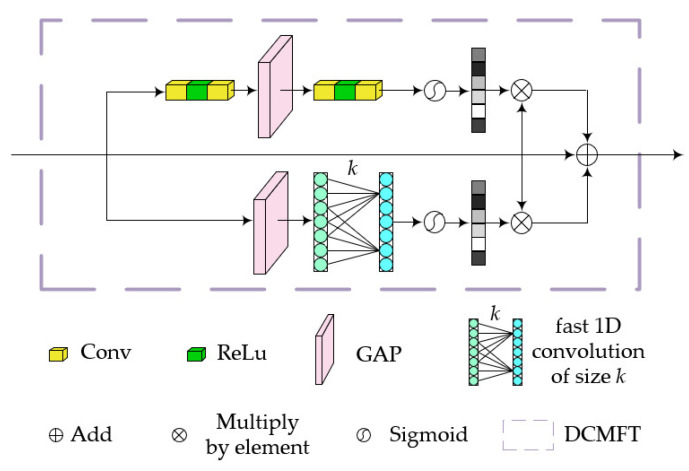
The structural details of DCMFT, with two branches divided to generate channel weights.

The U-Net [28] architecture introduced in 2015 is a variant of Fully Convolutional Networks [29], which are widely adopted in the field of semantic segmentation. The remarkable performance of U-Net is attributed to the inclusion of skip connections. However, in the original U-Net, the simplicity of the transmission structure leads to losses in the transmission of multi-scale contextual information. Therefore, to more effectively transmit the flow of information, especially during the downsampling process in encoding, we focused on optimizing the skip connections and proposed DCMFT, as illustrated in Figure 3.

Skip connections consist of two branches. One branch includes a channel attention module that aggregates features through convolution, PReLu activation, and global average pooling. The other branch generates channel weights by performing fast 1D convolutions with a size of k, where k is adaptively determined based on the channel dimension *C*, as expressed in the following equation:

(1) $$k=\varphi \left(C\right)={\left|\frac{log_{2}\left(C\right)}{\gamma }+\frac{b}{\gamma }\right|}_{odd},$$

where the parameters b and γ are the settings for the mapping function, with values of 1 and 2, respectively. ${\left|t\right|}_{odd}$ represents the nearest odd value to t. Subsequently, we introduce a side-branch for cross-layer connections using the generated weights. Finally, these two branches are fused to transmit information to the decoding part.

These enhancements to the U-Net framework improve its ability to capture multi-scale contextual information and ensure efficient information transfer within the skip connections.

### 3.2. iAFF

**Figure 4:**
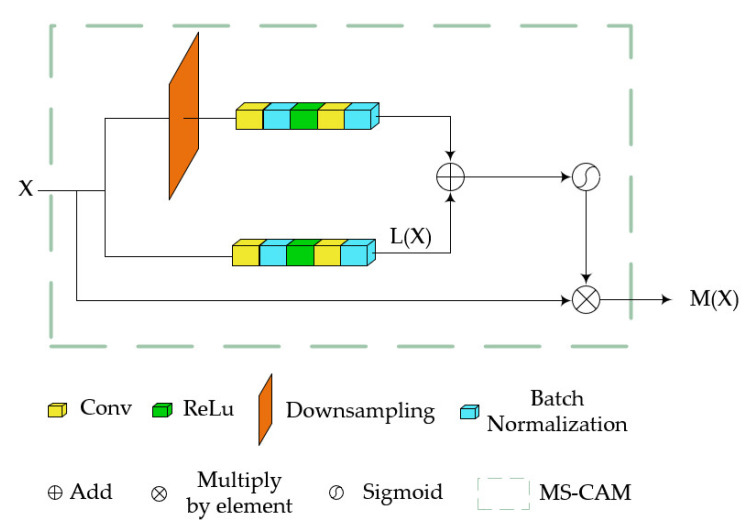
The structural details of MS-CAM, which constitute the weight generation component of the iAFF module.

To enhance the correlation between features at different stages, we have introduced the attention feature fusion mechanism, which is located between the first-stage and second-stage encoder–decoder in the corresponding layer. Its structure is shown in Figure 4. Typically, simple feature fusion can be achieved through addition or concatenation. However, this approach fixes the fusion weights. Therefore, we employ a selection mechanism to dynamically generate fusion weights based on two input features.

First, we will focus on the weight generation part, which is a multi-scale channel attention module showing in Figure 4. It changes the feature scale by the downsampling operation and then aggregates features using broadcast addition. Finally, it multiplies the result with the original input elements using the Sigmoid activation function to obtain the weights. Assuming the input $X\in {\mathbb{R}}^{C\times H\times W}$, the expression for the original scale branch is as follows:

(2) $$L\left(X\right)=B\left(C_{2}\left(R\left(B\left(C_{1}\left(X\right)\right)\right)\right)\right),$$

where $C_{1}$ and $C_{2}$ refer to convolution operations, B represents batch normalization processing, and R represents the ReLU activation function. The overall output is then calculated as

(3) $$M\left(X\right)=X*Sigmoid\left(L\left(d\left(X\right)\right)+L\left(X\right)\right),$$

in which d represents downsampling operation. For the weight $M\left(X\right)$, it will be applied to two features to be fused; we assume two input features, X and Y. The expression for one fusion process is as follows:

(4) $$\psi \left(X,Y\right)=M\left(X+Y\right)*X+\left(1-M\left(X+Y\right)\right)*Y.$$

**Figure 5:**
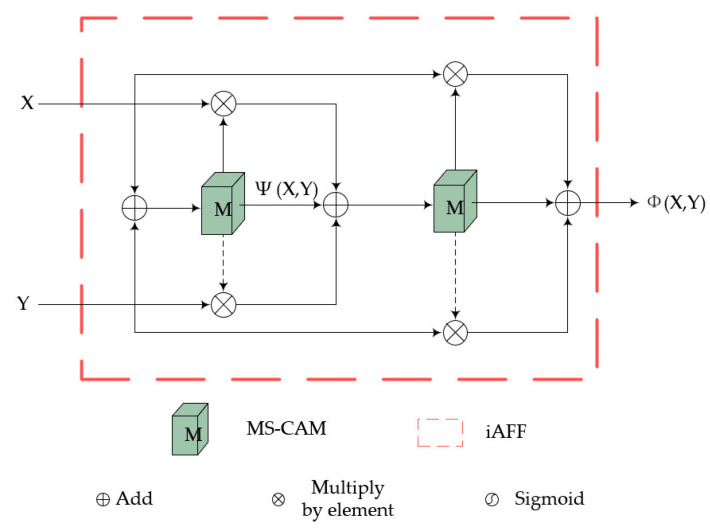
The overall structure of iAFF involves the iterative use of MS-CAM twice. The first input to MS-CAM is the sum of X and Y, and the second input is the output of the first iteration.

The entire process iterates twice to ensure the comprehensive fusion of input features, as shown in Figure 5, thereby achieving feature transfer from the lower stage to the upper stage.

### 3.3. SAM and ORSNet

The two modules adopt the structure used in previous research [16]. The SAM serves two primary functions: first, it utilizes the marine snow feature maps generated by the encoder–decoder module for snow removal, and second, it enhances the feature maps of marine snow using the results after snow removal. The enhanced feature maps are then concatenated with the untreated input of the next stage to ensure the full utilization of the marine snow features extracted in the previous stage. The ORSNet does not involve any downsampling operations and is composed of a concatenation of multiple channel attention modules, with the aim of preserving the details of the final stage image output.

### 3.4. Loss Function

The entire network structure employs a staged design, with each stage gradually deepening and producing its respective output. As a result, the overall loss function needs to encompass all losses from the three stages for holistic optimization of the entire marine snow removal network. The constructed loss function consists of three components: Charbonnier loss [30], Edge loss, and multi-scale structural similarity [31], expressed as follows:

(5) $$L=\overset{3}{\underset{P=1}{\sum }}[\sqrt{\|X_{P}-Y{\|}^{2}+\varepsilon^{2}}+\lambda \sqrt{\|\Delta \left(X_{P}\right)-\Delta \left(Y\right){\|}^{2}+\varepsilon^{2}}+\mu S\left(X,Y\right)],$$

where Y represents the ground truth image, X represents the image after snow removal at each stage, and Δ represents the Laplacian operator. In the first two loss terms, E is a constant set to 10^−3^, which ensures that gradients close to zero are not too small, preventing gradient vanishing. Simultaneously, the square root symbol constrains gradients far from zero from becoming too large, avoiding the problem of gradient explosion. The final term, $S\left(X,\;Y\right)$, represents multi-scale structural similarity, an improvement based on SSIM [32]. The general expression for SSIM is as follows:

(6) $$SSIM\left(X,Y\right)=l{\left(X,Y\right)}^{\alpha }*c{\left(X,Y\right)}^{\beta }*s{\left(X,Y\right)}^{\gamma },$$

where α, β, and γ are typically constants. In Formula (6), $l\left(X,Y\right)$ is used to estimate luminance, $c\left(X,\;Y\right)$ to estimate contrast, and $s\left(X,\;Y\right)$ to estimate structural similarity. Their expressions can refer to (7)–(9).

(7) $$l\left(X,Y\right)=\frac{2\mu_{x}\mu_{y}+c_{1}}{\mu_{x}^{2}+\mu_{y}^{2}+c_{1}},$$

(8) $$c\left(X,Y\right)=\frac{2\sigma_{x}\sigma_{y}+c_{2}}{\sigma_{x}^{2}+\sigma_{y}^{2}+c_{2}},$$

(9) $$s\left(X,Y\right)=\frac{\sigma_{xy}+c_{3}}{\sigma_{x}\sigma_{y}+c_{3}},$$

where $\mu_{x}$ represents the pixel average of image X, $\sigma_{x}$ is the standard deviation of the pixels in image X, and $\sigma_{xy}$ is the covariance between the pixels of image X and image Y.$\;c_{1},c_{2}$, and $c_{3}$ are constants used to prevent the denominator from being zero.

**Figure 6:**
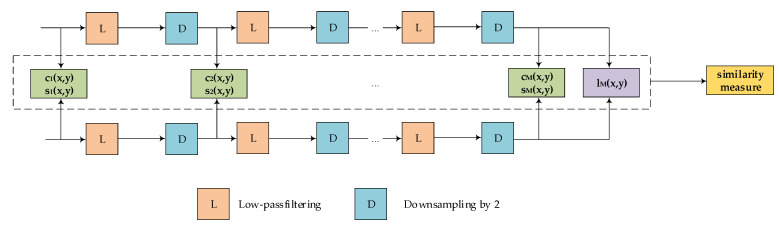
MS-SSIM computation flowchart. Calculate initial parameters $c_{1},s_{1}$ for the original image. Apply low-pass filtering and downsampling to compute parameters $c_{2},s_{2}$ for a one-level reduced image. Iterate to determine parameters $l_{m}$ for the minimum-sized image. Synthesize all parameters to obtain MS-SSIM.

Single-scale methods are limited to specific settings, neglecting the perceptibility of image details at various resolutions and viewing distances. Based on this, MS-SSIM is obtained by iteratively applying low-pass filtering and 1/2 downsampling, and finally concatenating results from different scales, with the ultimate expression as follows:

(10) $$S\left(X,Y\right)={\left[l\left(X,Y\right)\right]}^{\alpha_{M}}*\overset{M}{\underset{j=1}{\prod }}{\left[c\left(X,Y\right)\right]}^{\beta_{j}}*{\left[s\left(X,Y\right)\right]}^{\gamma_{j}},$$

in which M represents the number of low-pass filtering and downsampling iterations, and the entire computation process is illustrated in Figure 6.

Finally, in the overall loss function (5), the parameters λ and μ control the relative importance of the three loss components. After comprehensive experimentation, they have been determined λ=0.05,  μ=0.01.

## 4. Experiments

### 4.1. Experiments Configuration

The hardware and software configurations for all experiments in this paper are as shown in Table 1.

### 4.2. Dataset

In this paper, we adopt the public marine snow dataset MSRB [33], which is currently only available as a part of our training dataset. This dataset is modeled based on the pixels of marine snow using observational data from underwater images and consists of 2300 image pairs. To further enrich the dataset, we expanded the data by randomly selecting a certain number of underwater snow-free images from other underwater image datasets, including UFO120 [34] and UIEB [35], and generated marine snow according to the modeling approach of MSRB. Eventually, the training set of the dataset we constructed consists of 4439 image pairs, and the test dataset consists of 747 image pairs.

### 4.3. Parameter Settings

For the input image size, we chose 384 × 384 pixels. The optimization of the network was performed using the Adam optimizer [36] with an initial learning rate of 4 × 10^−5^ and a minimum learning rate of 1 × 10^−6^. We implemented a step-wise learning rate reduction strategy, set the batch size to 4, and conducted a total of 100 training epochs.

### 4.4. Comparative Experiments

To assess the model’s performance, following the evaluation method by Li et al. [37], we compared the trained model with other mainstream algorithms, using metrics such as the peak signal-to-noise ratio [38], structural similarity [32], mean squared error [39], model size, and inference speed. 

**Figure 7:**
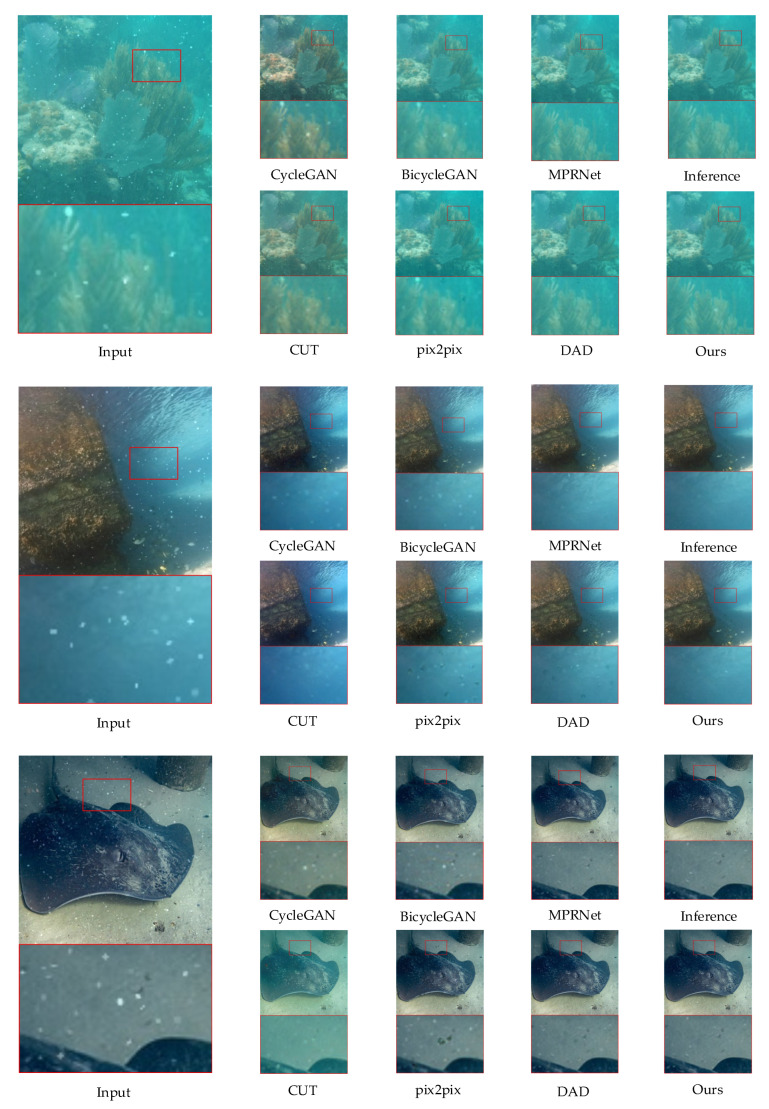
Comparison of marine snow removal effects across different methods. It can be observed that our method maintains the original image’s color tones while achieving a notably effective marine snow removal.

Qualitative comparison: In Figure 7, we present the results of different methods on a pre-processed dataset. It is evident that both CycleGAN and CUT [25] exhibit pronounced color deviations and retain remnants of marine snow. While pix2pix [24] and BicycleGAN [26] exhibit less severe color deviations than the former two, the fading of marine snow traces is still relatively minor, with some remnants persisting, though less prominently. 

Additionally, a closer examination of the magnified details reveals that the processed images from these four methods suffer from edge blurring. This implies that their snow removal capabilities come at the expense of image quality.

Regarding the results for DAD [40], despite its superior performance among non-targeted methods, the details reveal that, although it successfully removes marine snow and some edge details are preserved, it leaves the marine snow locations unfilled and results in the appearance of black spots, which also appear in the results of pix2pix. From a visual perspective, our proposed method appears to be optimal, a conclusion supported by the subsequent metric analyses.

Quantitative comparison: In addition to qualitative assessments, we conducted an exhaustive quantitative analysis to comprehensively evaluate the performance of various methods. Table 2 presents the quantitative comparison results regarding the de-snowing capabilities of the examined networks. Notably, our method exhibits significantly superior de-snowing efficacy compared to its counterparts, achieving a remarkable PSNR of 38.9251 dB. However, both CycleGAN and CUT struggle with suboptimal de-snowing outcomes due to global color discrepancies, yielding PSNR values of only 25.3431 dB and 26.2229 dB, respectively.

In contrast, pix2pix and BicycleGAN showcase relatively improved performance metrics, attaining PSNR values of 30.9850 dB and 30.4210 dB. The incorporation of two Markov discriminators within DAD’s network architecture enhances perceptual quality, resulting in an impressive PSNR of 34.2794 dB.

In the latter part of Table 2, we present a comparative analysis of various algorithms with respect to model size, and real-time performance. The real-time efficiency is quantified using Frames Per Second (FPS). The results illustrate that while our proposed method may not achieve the fastest inference speed, lagging behind the swiftest pix2pix inference by 6 FPS, it still stands at a respectable 10.44 FPS among all the compared methods. Notably, the entire network is compact, measuring a mere 45.23 MB. In contrast, the less effective DAD processes at a sluggish 2.26 FPS with a colossal model size of 813.70 MB. Consequently, it is evident that our approach, with its superior snow removal efficacy, also excels in inference speed.

### 4.5. Ablation Experiments

In this section, we describe the conducted comprehensive ablation experiments to validate the role of the improved module within the overall network. Specifically, we examine the contributions of DCMFT, iAFF, and the final loss function. Additionally, given that our proposed method is a multi-stage network, we assess the effectiveness of this multi-stage structure by obtaining outputs at each stage. 

Table 3 presents the specific results of the ablation experiments. Analyzing each corresponding phase reveals PSNR improvement increments of 0.6140 dB, 0.9840 dB, and 0.4143 dB for the first, second, and third stages, respectively. The smaller improvement in the third stage is primarily attributed to the concentration of the network’s feature propagation and fusion modules in the preceding phases. Furthermore, in each set of ablation experiments, there is a consistent improvement of approximately 1–2 dB between adjacent stages, validating the effectiveness of the multi-stage progressive restoration network structure.

**Figure 8:**
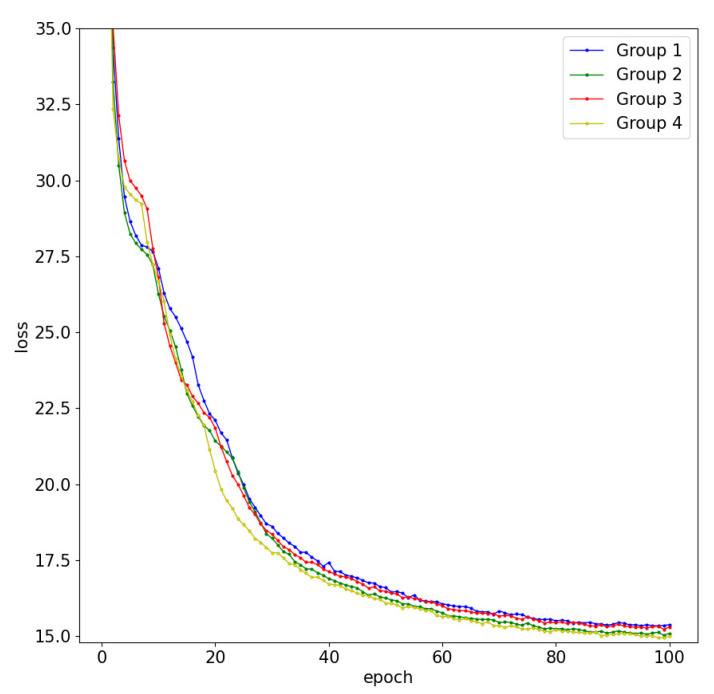
Loss functions during training processes in various ablation experiment groups.

In addition, we visually represented the variations in the loss functions during each training epoch for the different ablation experiments as shown in Figure 8. Observing the line chart, it becomes evident that the introduction of the iAFF module led to a notable reduction in training loss compared to the baseline. Subsequently, the incorporation of MS_SSIM, while contributing an additional loss term, resulted in a slight improvement over the baseline, despite an expected increase in training loss. Finally, with the integration of DCMFT, the overall loss curve reached its optimal point among all the ablation experiments, exhibiting the fastest convergence and ultimately achieving the minimum training loss.

## 5. Conclusions

In this paper, we introduce the concept of multi-stage processing and propose the MP-MSRN for the task of marine snow removal. Within the network architecture, we establish the DCMFT to minimize the loss of features during the encoding process. Additionally, the introduction of the iAFF facilitates the fusion of features, maximizing the utilization of marine snow features extracted at different stages. Furthermore, we devise a novel loss function incorporating MS-SSIM to expedite the convergence speed during training. Finally, compared with existing marine snow removal methods, our network achieves a comprehensive result by iteratively extracting and enhancing marine snow features at each stage, demonstrating its superior performance through both qualitative and quantitative analyses. Future work will focus on the real-time removal of marine snow from video frames. One promising approach involves leveraging information between adjacent frames while addressing the challenge of minimizing the impact of background motion.

## Figures and Tables

**Table 1:** The software and hardware configurations used for comparison and ablation experiments.

Hardware		Software	
Divices	Model/Size	Tool Stack	Version
CPU	64 Intel(R) Xeon(R) Gold 6346	OS	Ubuntu 18.04
RAM	256 GB	Python	3.9
GPU	Nvidia GTX3090	CUDA	11.4
\	\	Pytorch	1.12

**Table 2:** Metrics of marine snow removal effects across different methods, with the best results highlighted in bold.

Method	PSNR (dB)	SSIM	MSE	Speed (FPS)	Model Size (MB)
CycleGAN [23]	25.3431	0.8382	348.3727	7.26	43.42
pix2pix [24]	30.9850	0.8846	68.8730	**16.58**	207.64
BicycleGAN [26]	30.4210	0.8653	159.4803	7.10	209.04
CUT [25]	26.2229	0.8440	287.0160	14.25	43.46
MPRNet [16]	38.5108	0.9747	14.2182	10.91	**41.97**
DAD [40]	34.2794	0.9257	41.4867	2.26	813.70
Ours	**38.9251**	**0.9761**	**13.4415**	10.44	45.23

**Table 3:** Result indicators for different groups in the ablation experiment. For the module part, we selected MPRNet as the baseline. iAFF is positioned between the first and second stages, serving to fuse features. Therefore, both stages one and two are selected in the ablation experiment.

Group	Module				Stage	PSNR (dB)	SSIM	MSE
	Baseline	iAFF	MS_SSIM	DCMFT				
1	√	\	\	\	1	35.1214	0.9581	26.3924
		\	\	\	2	36.2208	0.9654	21.5644
		\	\	\	3	38.5108	0.9747	14.2182
2		√	\	\	1	35.3099	0.9600	25.6271
		√	\	\	2	36.6715	0.9676	19.5356
		\	\	\	3	38.6133	0.9753	14.1122
3		√	√	\	1	35.4822	0.9607	24.7388
		√	√	\	2	36.7619	0.9678	19.1418
		\	√	\	3	38.7733	0.9756	13.8419
4		√	√	√	1	35.7354	0.9621	23.6104
		√	√	√	2	37.2048	0.9690	17.7378
		\	√	\	3	38.9251	0.9761	13.4415

## Data Availability

Data are contained within the article.
